# En Bloc Right Upper Bilobectomy For a Patient With Incomplete Minor Fissure and Multiple Anomalies of the Right Superior Pulmonary Vein

**DOI:** 10.7759/cureus.70080

**Published:** 2024-09-24

**Authors:** Masakazu Matsuda, Hiroshi Mizuuchi, Kensaku Ito, Hidenori Kouso

**Affiliations:** 1 Department of Surgery, Oita Red Cross Hospital, Oita, JPN; 2 Department of Thoracic Surgery, Shimonoseki City Hospital, Shimonoseki, JPN; 3 Department of Thoracic Surgery, Oita Red Cross Hospital, Oita, JPN

**Keywords:** incomplete interlobar fissure, non-small cell lung cancer, pulmonary vein variation, three-dimensional computed tomography, uniportal video-assisted thoracoscopic surgery

## Abstract

Variations in the anomaly of the right superior pulmonary vein can lead to perioperative complications if misunderstood. Additionally, incomplete fissures increase the complexity of performing pulmonary anatomical resection. Therefore, accurate preoperative anatomical assessment using three-dimensional computed tomography is crucial for ensuring safe surgery. We herein report a case of en bloc right upper bilobectomy via uniportal video-assisted thoracic surgery in a patient with an incomplete minor fissure and a complex anomaly of the right superior pulmonary vein.

## Introduction

Several patterns of variation in the right superior pulmonary vein have been reported [1], although rare anomalies also exist. Misunderstanding these anatomical variations can result in complications such as intraoperative bleeding and incorrect anatomic resection. Furthermore, an incomplete fissure can complicate anatomical resection by potentially increasing the risk of postoperative prolonged air leak [2]. Therefore, accurate preoperative evaluation using three-dimensional computed tomography (3D-CT) is essential to ensure safe anatomical resection. We herein present a challenging case of en bloc right upper bilobectomy in a patient with primary lung cancer located within an incomplete minor fissure, accompanied by multiple anomalies of the right superior pulmonary vein.

## Case presentation

**Figure 1:**
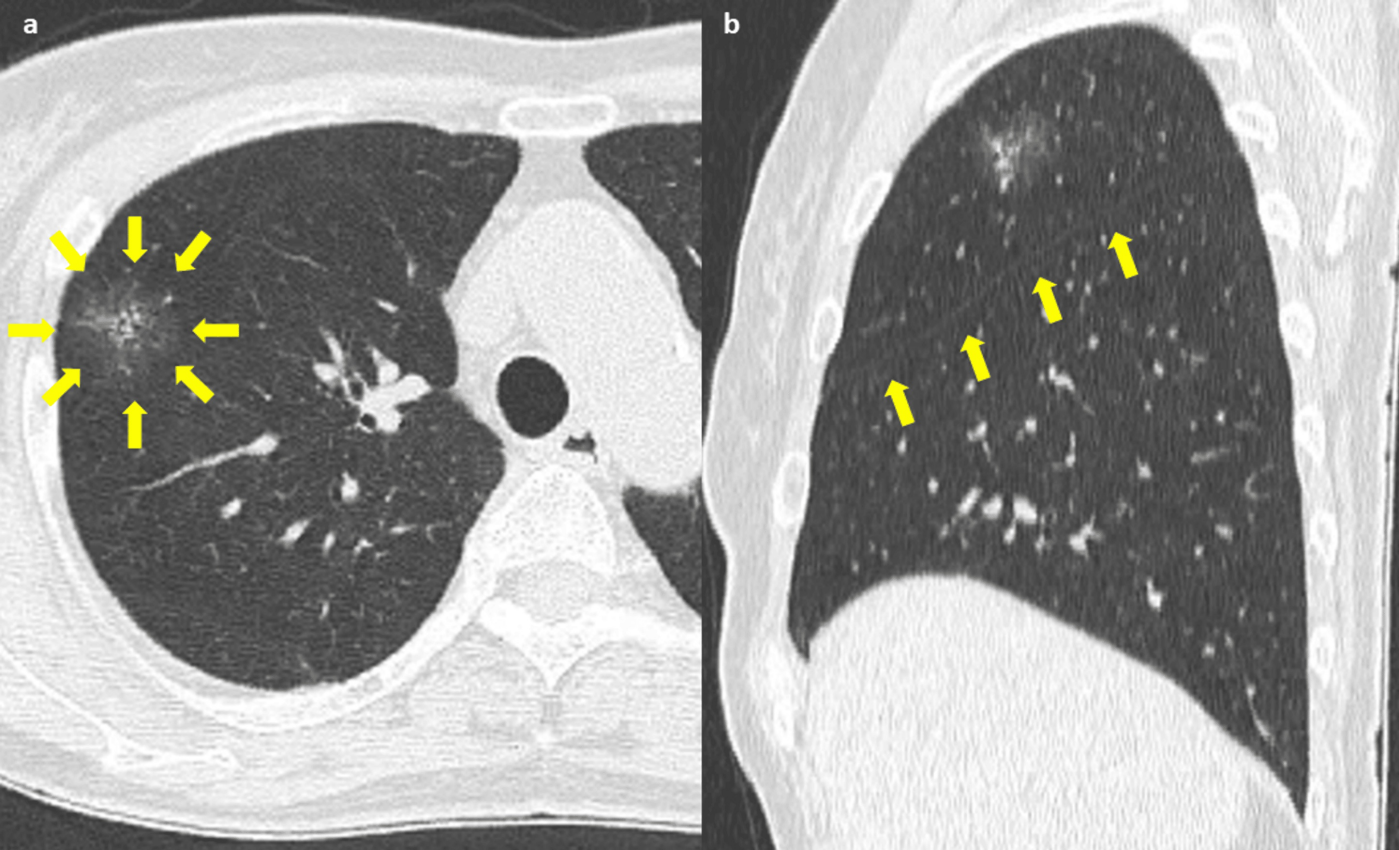
Chest computed tomography images (axial and sagittal views) (a) A mixed ground-glass nodule, 2.0 cm and 0.7cm in total and solid size, located between the right upper and middle lobes. (b) Yellow arrows indicate the major fissure. The minor fissure is barely visible.

**Figure 2:**
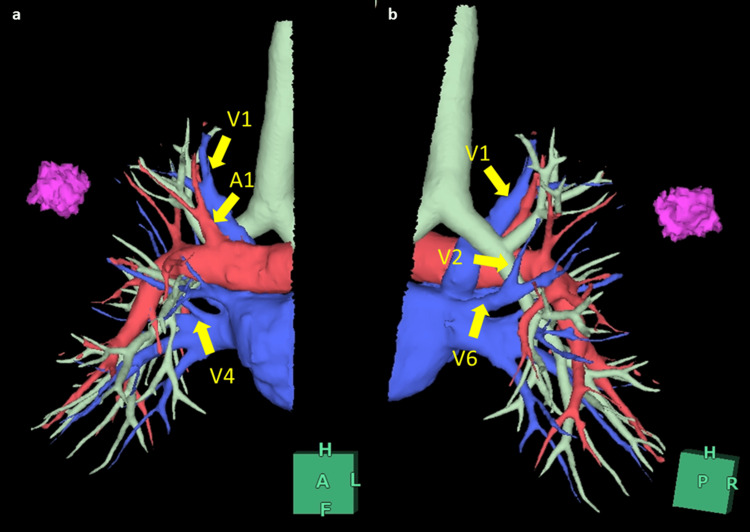
Preoperative estimation of pulmonary vessels and bronchi using three-dimensional computed tomography (a) The anomalous V1 runs behind the superior trunk of the pulmonary artery (A1), and the V4 drains into the inferior pulmonary vein. B3, A3, and V3 are absent, and segment 3 is aplastic. (b) The aberrant V2 drains into the V6 behind the right bronchus intermedius.

A 74-year-old woman was referred to our department after a CT scan, which, initially performed for an inguinal hernia, revealed a ground-glass nodule in the right upper lobe. After three years, a follow-up CT showed that the nodule had grown to a diameter of 2.0 cm (solid component was 0.7 cm) and was situated at the incomplete minor fissure (Figure 1). The clinical stage was assessed as Stage IA1 (T1aN0M0) primary lung cancer, and surgery was recommended for both diagnostic and therapeutic purposes. Preoperative 3D-CT revealed multiple anomalies in the superior pulmonary vein, including an unusual course of the V1 running between the pulmonary artery and the right main bronchus, the V2 draining into the V6 behind the right bronchus intermedius, and the V4 draining into the right inferior pulmonary vein (Figure 2). Due to agenesis of segment 3 (absence of B3, A3, and V3), the right upper and middle lobes together constituted 23% of the total lung volume. Considering these factors, a combined resection of the right upper and middle lobes was planned to ensure a sufficient surgical margin.

The surgery was performed using a uniportal video-assisted thoracic surgery approach, with a 4-cm incision along the anterior axillary line at the fifth intercostal space. The superior trunk of the pulmonary artery (A1) was transected using an endostapler, followed by the identification and separation of the dorsal V1. After dissecting the right upper lobe bronchus, the aberrant V2 behind the right main bronchus, which drained into the V6, was sealed and cut using an ultrasonic device. Subsequent steps included the dissection of A2, A5, V5, V4, and A4, followed by creation of the posterior major fissure with an endostapler. Finally, the right middle lobe bronchus was separated, completing the en bloc resection of the upper and middle lobes (Video 1). The postoperative course was uneventful.

**Video 1:** Uniportal VATS en bloc right upper bilobectomy for lung cancer located at an incomplete fissure En bloc resection of right upper and middle lobes was completed via uniportal VATS because the tumor was located at an incomplete minor fissure. Multiple variations of the right superior pulmonary vein exist. V1 ran behind the superior trunk of the pulmonary artery (A1), V2 drained into V6 behind the right bronchus intermedius, and V4 drained into the right inferior pulmonary vein. VATS: Video-assisted thoracoscopic surgery

**Figure 3:**
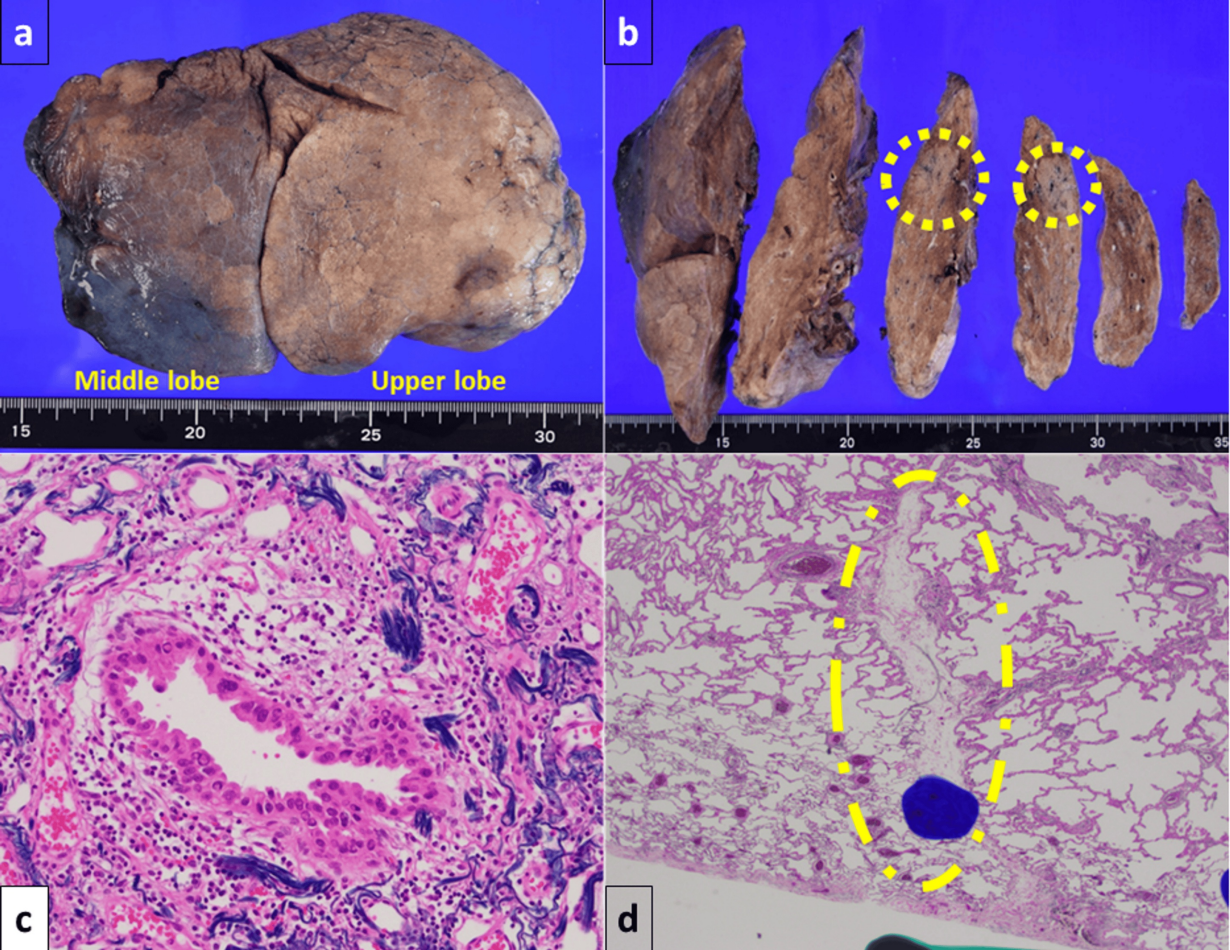
Macroscopic and microscopic findings of the resected specimen (a) Macroscopic image of the resected specimen showing the incomplete minor fissure between the right upper and middle lobes. (b) The lesion is located at the incomplete minor fissure. (c, d) The tumor is an acinar adenocarcinoma with surrounding lepidic growth, measuring 2.1 cm in overall diameter and 0.7 cm in invasive diameter. The lesion invades beyond the incomplete interlobar pleura, leading to a diagnosis of T2aN0M0 Stage IB.

The pathological diagnosis was adenocarcinoma. The nodule measured 2.1 cm in diameter, with an invasive component of 0.7 cm. Given the lesion’s location across the incomplete minor fissure, the patient was diagnosed with pT2aN0M0, Stage IB lung cancer (Figure 3). She subsequently underwent two years of postoperative adjuvant chemotherapy with tegafur/uracil. At the time of this writing, the patient had been under observation for two years post-treatment with no signs of recurrence.

## Discussion

Anatomical variations of the right superior pulmonary vein are primarily classified into four types: two anterior with central types (Ia and Ib), the central type, and the anterior type [1]. However, rare anomalies not listed in the report by Shimizu et al. also exist [1]. As shown in Table 1, 12 cases, including ours, have been reported to have the V1 running dorsally to the right main pulmonary artery [3-13]. Overlooking this anomaly can lead to a significant risk of injuring the dorsal pulmonary veins and causing bleeding during transection of the superior trunk. Therefore, it is crucial to identify such anomalies preoperatively.

**Table 1:** Reported cases of the right SPV behind the main PA SPV, superior pulmonary vein; PA, pulmonary artery; PV, pulmonary vein; RBI, right bronchus intermedius; IPV, inferior pulmonary vein; TB, tracheal bronchus; RUL, right upper lobectomy; RLL, right lower lobectomy, RUML, right upper and middle lobectomy

Case	Age/Sex	Other PV anomalies	Bronchial anomaly	Surgery	References
1	58/F	-	TB	RUL	Yurugi et al. 2012 [3]
2	54/M	-	-	RUL	Sumitomo et al. 2016 [4]
3	74/M	-	B1+3 and B2 branched separately	RLL	Ichiki et al. 2017 [5]
4	66/F	-	TB	RUL	Nakamura et al. 2017 [6]
5	64/F	aberrant V2 behind RBI	-	RUL	Otsuki et al. 2019 [7]
6	66/F	aberrant V2 behind RBI	Absence of the bronchus inter medius	RUL	Huang et al. 2020 [8]
7	75/F	-	-	RUL	Kamimura et al. 2020 [9]
8	79/F	aberrant V2 behind RBI	TB	RUL	Mun et al. 2020 [10]
9	66/F	-	-	RUL	Wang et al. 2020 [11]
10	44/F	V4+5 into IPV	B1 and B2+3 branched separately	RUL	Wang et al. 2021 [12]
11	70s/M	-	-	RUL	Uchida et al. 2022 [13]
12	76/F	aberrant V2 behind RBI V4 into IPV	-	RUML	Matsuda et al. 2024 (current case)

These anomalies are often associated with other bronchial or pulmonary vein anomalies. Among the cases reviewed, three were associated with tracheal bronchus, and three involved the V2 draining into the inferior pulmonary vein or the left atrium behind the right bronchus intermedius. Conversely, recognizing these anomalies should prompt the consideration of an anomalous V1. In our case, multiple anomalies of the pulmonary veins draining the right upper and middle lobes were identified preoperatively using 3D-CT. This comprehensive understanding enabled the successful completion of an en bloc right upper bilobectomy through uniportal video-assisted thoracic surgery without confusion or complications.

Additionally, it is essential to consider the appropriateness of bilobectomy for primary lung cancer with adjacent lobe invasion due to an incomplete fissure. Andreetti et al. reported that direct invasion into the adjacent lobe through an incomplete fissure, a tumor size of less than 5 cm, and the absence of lymph node metastasis are favorable prognostic factors [14]. In our case, a right upper bilobectomy was performed to ensure adequate margins for a 2.0-cm part solid ground-glass nodule located between the upper and middle lobes in an incomplete lobulation. Furthermore, 3D-CT volumetry revealed that the volume loss after the right upper bilobectomy was approximately 23% due to the agenesis of S3, making the invasiveness of the procedure tolerable for the patient. Consequently, her postoperative course was uneventful, and she was able to promptly begin adjuvant chemotherapy.

## Conclusions

We have herein reported a challenging case involving multiple pulmonary venous anomalies and primary lung cancer located in an incomplete minor fissure. Preoperative 3D-CT proved useful in estimating individual anatomy, including pulmonary vessels and lung volume, thus facilitating the planning of a safe surgical procedure.
